# Effects of the protein kinase inhibitors wortmannin and KN62 on cellular radiosensitivity and radiation-activated S phase and G1/S checkpoints in normal human fibroblasts

**DOI:** 10.1038/sj.bjc.6690793

**Published:** 1999-11

**Authors:** L Enns, D Murray, R Mirzayans

**Affiliations:** Department of Oncology, University of Alberta, Cross Cancer Institute, Edmonton, Alberta, T6G 1Z2 Canada

**Keywords:** wortmannin, KN62, ionizing radiation, radiosensitivity, cell cycle checkpoint, p53, *p21^WAF1^*

## Abstract

Wortmannin is a potent inhibitor of phosphatidylinositol (PI) 3-kinase and PI 3-kinase-related proteins (e.g. ATM), but it does not inhibit the activity of purified calmodulin-dependent protein kinase II (CaMKII). In the present study, we compared the effects of wortmannin and the CaMKII inhibitor KN62 on the response of normal human dermal fibroblast cultures to γ radiation. We demonstrate that wortmannin confers a phenotype on normal fibroblasts remarkably similar to that characteristic of cells homozygous for the *ATM* mutation. Thus wortmannin-treated normal fibroblasts exhibit increased sensitivity to radiation-induced cell killing, lack of temporary block in transition from G1 to S phase following irradiation (i.e. impaired G1/S checkpoint), and radioresistant DNA synthesis (i.e. impaired S phase checkpoint). Wortmannin-treated cultures display a diminished capacity for radiation-induced up-regulation of p53 protein and expression of *p21^WAF1^*, a p53-regulated gene involved in cell cycle arrest at the G1/S border; the treated cultures also exhibit decreased capacity for enhancement of CaMKII activity post-irradiation, known to be necessary for triggering the S phase checkpoint. We further demonstrate that KN62 confers a radioresistant DNA synthesis phenotype on normal fibroblasts and moderately potentiates their sensitivity to killing by γ rays, without modulating G1/S checkpoint, p53 up-regulation and *p21^WAF1^* expression following radiation exposure. We conclude that CaMKII is involved in the radiation responsive signalling pathway mediating S phase checkpoint but not in the p53-dependent pathway controlling G1/S checkpoint, and that a wortmannin-sensitive kinase functions upstream in both pathways. © 1999 Cancer Research Campaign


*British Journal of Cancer* (1999) **81**(6), 959–965
© 1999 Cancer Research Campaign
Article no. bjoc.1999.0793

### Effects of the protein kinase inhibitors wortmannin and KN62 on cellular radiosensitivity and radiation-activated S phase and G1/S checkpoints in normal human fibroblasts

**L Enns, D Murray and R Mirzayans**

Department of Oncology, University of Alberta, Cross Cancer Institute, Edmonton, Alberta, T6G 1Z2 Canada

**Summary** Wortmannin is a potent inhibitor of phosphatidylinositol (PI) 3-kinase and PI 3-kinase-related proteins (e.g. ATM), but it does not inhibit the activity of purified calmodulin-dependent protein kinase II (CaMKII). In the present study, we compared the effects of wortmannin and the CaMKII inhibitor KN62 on the response of normal human dermal fibroblast cultures to γ radiation. We demonstrate that wortmannin confers a phenotype on normal fibroblasts remarkably similar to that characteristic of cells homozygous for the *ATM* mutation. Thus wortmannin-treated normal fibroblasts exhibit increased sensitivity to radiation-induced cell killing, lack of temporary block in transition from G1 to S phase following irradiation (i.e. impaired G1/S checkpoint), and radioresistant DNA synthesis (i.e. impaired S phase checkpoint). Wortmannin-treated cultures display a diminished capacity for radiation-induced up-regulation of p53 protein and expression of $p21^{WAF1}$, a p53-regulated gene involved in cell cycle arrest at the G1/S border; the treated cultures also exhibit decreased capacity for enhancement of CaMKII activity post-irradiation, known to be necessary for triggering the S phase checkpoint. We further demonstrate that KN62 confers a radioresistant DNA synthesis phenotype on normal fibroblasts and moderately potentiates their sensitivity to killing by γ rays, without modulating G1/S checkpoint, p53 up-regulation and $p21^{WAF1}$ expression following radiation exposure. We conclude that CaMKII is involved in the radiation responsive signalling pathway mediating S phase checkpoint but not in the p53-dependent pathway controlling G1/S checkpoint, and that a wortmannin-sensitive kinase functions upstream in both pathways. © 1999 Cancer Research Campaign

**Keywords:** wortmannin; KN62; ionizing radiation; radiosensitivity; cell cycle checkpoint; p53; $p21^{WAF1}$

Proliferating human cells exposed to ionizing radiation display a variety of complex cellular responses, including a delay in normal progression through the cell cycle at several checkpoints. These checkpoints are regulated by mitogenic signal transduction pathways and feedback loops which are themselves coordinately regulated by external signals. In many cell types, the signalling pathway which mediates transitory blockage of progression from G1 to S phase following genotoxic stresses is dependent on the p53 tumour suppressor protein. Irradiation of normal human fibroblast cultures, for example, results in activation of p53 via a post-translational stabilization mechanism. This, in turn, leads to increased expression of the $p21^{WAF1}$ gene, which encodes an inhibitor of cyclin-dependent kinases that are required for G1/S progression (El-Deiry et al, 1993; Brugarolas et al, 1995). Radiation-induced activation of p53 has been reported to be mitigated by calphostin C, a specific inhibitor of protein kinase C, suggesting the involvement of this kinase in the p53 pathway (Khanna and Lavin, 1993). Using pharmacological inhibitors of different protein kinases, we provided evidence that the radiation-responsive signalling cascade regulating replicative DNA synthesis (i.e. S phase checkpoint) in normal human fibroblasts utilizes $Ca^{2+}$/calmodulin-dependent protein kinase II (CaMKII) but not protein kinase C (Mirzayans et al, 1995*a*). The role of CaMKII in the p53-signalling pathway remains to be elucidated.

Ataxia telangiectasia (AT) is a recessive human genetic disease featuring immunological, neurological and developmental abnormalities, cancer proneness and hypersensitivity to ionizing radiation (Sedgwick and Boder, 1991; Lavin, 1993). Cells cultured from AT patients exhibit impaired induction of p53 and defects in all cell cycle checkpoints normally seen at early times post-irradiation (Kastan et al, 1992; Beamish and Lavin, 1994). A gene mutated in AT has been cloned and designated *ATM* (Savitsky et al, 1995). One of the functional domains of the ATM protein has significant homology with the lipid kinase domain of phosphatidylinositol (PI) 3-kinase (Savitsky et al, 1995), a cytoplasmic signal transducer that participates in mitogenesis, cell transformation and other cellular processes involving protein tyrosine kinases. PI 3-kinase and related proteins such as ATM and DNA-dependent protein kinase (DNA-PK) play important roles in vital biological processes including cellular responses to genotoxic stress (Taylor, 1998). Accordingly, there is a great deal of interest in pharmacological inhibitors of these kinases not only as a tool for elucidating the function of the target proteins, but also for exploring the possible clinical utility of such inhibitors as potentiators of the cytotoxicity of antineoplastic agents.

The purpose of the present study was to assess the effects of two protein kinase inhibitors, wortmannin and KN62, on the response of normal human dermal fibroblast cultures to $^{60}$Co γ radiation in terms of cell survival and activation of S phase and G1/S checkpoints. Wortmannin is an inhibitor of PI 3-kinase and PI

*Received 6 January 1999*
*Revised 14 May 1999*
*Accepted 19 May 1999*

*Correspondence to:* R Mirzayans

3-kinase-related proteins (Powis et al, 1994; Hartley et al, 1995; Banin et al, 1998; Cliby et al, 1998), but it does not inhibit the activity of purified CaMKII (Nakanishi et al, 1992). KN62, on the other hand, is a potent and specific inhibitor of CaMKII (Tokumitsu et al, 1990). We report that normal fibroblasts treated with wortmannin exhibit an AT-like phenotype, with regards to increased sensitivity to radiation cytotoxicity, mitigated G1/S checkpoint, and radioresistant DNA synthesis. Wortmannin-treated cultures also exhibit a diminished capacity for both up-regulation of p53 protein and enhancement of CaMKII activity following radiation exposure. We further demonstrate that the CaMKII inhibitor, KN62, abrogates the radiation-induced S phase checkpoint and moderately increases radiosensitivity in normal fibroblasts. Unlike wortmannin, however, KN62 exerts no significant effect on the G1/S checkpoint and on p53 up-regulation induced by γ rays.

### MATERIALS AND METHODS

### Cells and culture conditions

The normal human dermal fibroblast strain GM38 was obtained from NIGMS Human Genetic Mutant Cell Repository (Camden, NJ, USA). Cells were cultivated in thymidine (dThd)-free Ham's F12 medium supplemented with 10% (v/v) fetal bovine serum, 1 mM L-glutamine, 100 IU $ml^{-1}$ penicillin G and 100 μg $ml^{-1}$ streptomycin sulphate in a 37°C chamber incubator providing a humidified atmosphere of 5% carbon dioxide in air.

### Radiation treatment

Exposure to $^{60}$Co γ radiation was performed in a Gammacell 220 unit as described (Mirzayans et al, 1995*b*).

### Chemicals

Wortmannin and 1-[*N,O*-bis(5-isoquinolinesulphonyl)-*N*-methyl-*L*-tyrosyl]-4-phenylpiperazine (KN62) were purchased from Biomol Research Laboratories, Inc. (Plymouth Meeting, PA, USA). Stock solutions of these compounds (10 mM) were prepared in dimethyl sulphoxide (DMSO) and stored at –70°C. To assess the effects of kinase inhibitors on the response of human cells to γ radiation, cultures were treated with each reagent for 1 h prior to irradiation and during post-irradiation incubation periods as indicated. Control cultures were incubated in medium containing 0.1% (v/v) DMSO.

### Clonogenic survival assay

Fibroblasts harvested in late logarithmic growth phase were plated out at ~$5 \times 10^5$ per 100-mm dish and incubated overnight. Cells were treated with a kinase inhibitor for 1 h, exposed to γ rays and incubated in the same medium for 10 h. Their reproductive capacity was determined using the feeder layer technique described previously (Mirzayans et al, 1989).

### DNA replicative synthesis assay

Cells were inoculated in 60-mm dishes ($10^5$ cells per dish) and incubated overnight in growth medium and for an additional 18–20 h in medium containing 180 Bq $ml^{-1}$ [*methyl*-$^{14}$C]dThd (stock specific activity, $2 \times 10^9$ Bq $mmol^{-1}$). After removal of the radioactive medium, cultures were incubated for 1 h in the presence or absence of a protein kinase inhibitor as indicated, exposed to γ rays (or left unirradiated), and incubated for 1.5 h in the same medium. Tritiated-dThd ($5.5 \times 10^5$ Bq $ml^{-1}$; specific activity, $3 \times 10^{12}$ Bq $mmol^{-1}$) was added to the culture medium during the last 30-min of the post-irradiation incubation period. Cells in each dish were then lysed in a 2% sodium dodecyl sulphate (SDS) solution and the amount of trichloroacetic acid (TCA)-insoluble radioactivity in each lysate was determined as described (Mirzayans et al, 1995*b*). The level of DNA synthesis was expressed as a percentage of the resulting $^3$H/$^{14}$C ratios for irradiated compared to unirradiated cultures.

### Measurement of S phase index

Cells were seeded on sterile glass coverslips (placed in 35-mm dishes) at ~$5 \times 10^4$ cells in a final volume of 2 ml of dThd-free medium. After incubation for 2 days, cultures were exposed to γ rays (or left unirradiated), incubated for various times in dThd-free medium and then pulse-labelled for 0.5 h in medium containing $3.7 \times 10^4$ Bq [*methyl*-$^3$H]dThd (specific activity, $2.4 \times 10^{11}$ Bq $mmol^{-1}$) per ml. Cultures were incubated with or without a kinase inhibitor for 1 h prior to irradiation and during the entire post-irradiation period. The percentage of cells in S phase was determined by in situ autoradiography as described (Mirzayans et al, 1995*b*).

### CaMKII assay

CaMKII activity in cellular extracts was determined by the method of Mayford et al (1995) with minor modifications. GM38 fibroblasts were seeded in 150-mm dishes ($1 \times 10^6$ cells per dish) and incubated for 2 days in growth medium. The cells were incubated in serum-free medium for 90 min and then exposed to γ radiation (or sham-treated). Cells were incubated in medium containing wortmannin (10 μM) or DMSO for 1 h prior to and during irradiation. After rinsing in ice-cold phosphate-buffered saline (PBS), the cells were scraped into suspension and lysed for 30 min at 4°C in a buffer containing 40 mM Tris–HCl (pH 7.4), 0.5 mM EGTA, 0.5 mM EDTA (pH 7.4), 200 mM sodium chloride, 1% NP-40, 10 μg $ml^{-1}$ leupeptin, 0.4 mM sodium molybdate,10 mM sodium pyrophosphate, 20 mM glycerophosphate, 0.1 mM phenylmethylsulphonyl fluoride (PMSF) and 2 mM 2-mercaptoethanol. CaMKII reaction mixture contained 50 mM HEPES (pH 7.0), 10 mM magnesium chloride, 100 μM bovine serum albumin, 100 μM ATP, 100 μCi $ml^{-1}$ [γ-32P]ATP, 2 mM EGTA, 1 mM β-mercaptoethanol, 0.5 μM protein kinase A (PKA) inhibitor, and 20 μM autocamtide-2. Enzyme reactions were carried out at 37°C for 2 min in a final volume of 100 μl. Thereaction was started by the addition of 10 μl of cell extract and terminated by the addition of 100 μl of ice-chilled 10% TCA. Precipitated protein was pelleted by centrifugation in a microfuge for 4 min. The supernatant was spotted onto phosphocellulose filter discs (Gibco-BRL, Burlington, ON, Canada), washed with water (three times, 10 min each), and dried. The amount of $^{32}$P incorporated into the substrate (autocamtide-2) was quantified by scintillation counting. Protein concentrations of cell extracts were determined by the Bradford assay (Bio-Rad Laboratories, Hercules, CA, USA).

© 1999 Cancer Research Campaign

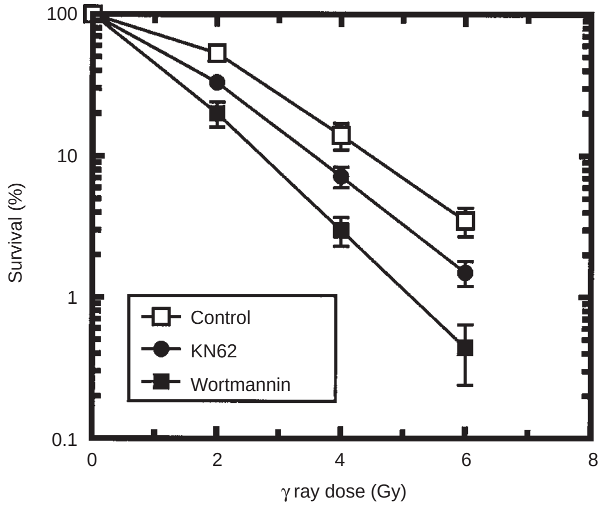

**Figure 1** Radiosensitization of GM38 fibroblasts by wortmannin and KN62. Cells were treated with either wortmannin or KN62, each at 10 μM; control cultures were incubated in medium containing 0.1% (v/v) DMSO. The cells were then exposed to γ rays, incubated in the same medium for 10 h and assayed for clonogenic survival as described in Materials and Methods. Bars, s.e.m.

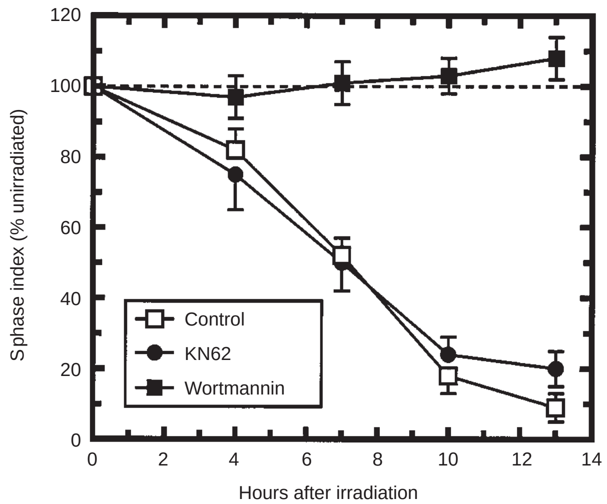

**Figure 2** Effects of wortmannin and KN62 on radiation-induced decreases in S phase index in GM38 fibroblasts. Cultures were incubated for 1 h either with or without a kinase inhibitor (10 μM). Following irradiation (10 Gy), the cells were incubated in the same medium for various times as indicated; [$^3$H]-dThd was added to the culture medium during the last 30 min. Cells were processed for autoradiography and the fraction of cells in S phase (i.e. those with heavily labelled nuclei) was determined. Each datum point represents the mean (± s.e.m.) of the values obtained in four samples from two independent experiments; at least 500 cells were counted for each sample

### Western blotting

GM38 fibroblasts were seeded in 100-mm dishes (~5 × $10^5$ per dish) and incubated in growth medium for 2 days. The cells were treated with a kinase inhibitor, exposed to γ rays, and incubated for 3 h. The cells were then centrifuged and resuspended in lysis buffer (62 mM Tris (pH 6.8)), 10% glycerol, 2% SDS, 5% β-mercaptoethanol, 0.003% bromophenol blue). Cellular proteins were separated by 10% SDS-polyacrylamide gel electrophoresis (SDS-PAGE) (30 μg protein loaded per lane) and transferred to Hybond-P membranes (Amersham Corp., Oakville, ON, Canada). The membranes were washed for 1 h in blocking buffer (5% non-fat dry Carnation milk in PBS), and incubated for 1 h in the same buffer containing a p53-specific monoclonal antibody (1801) or a p21-specific monoclonal antibody (Santa Cruz Biotechnology Inc., Santa Cruz, CA, USA). Antibody reactions were visualized by the Amersham chemiluminescence procedure using a horse-radish peroxidase-conjugated goat anti-mouse IgG as the secondary antibody (Santa Cruz Biotechnology Inc.), and quantified by a digitized image analyser (Palcic and Jaggi, 1990).

### Northern blotting

RNA was isolated from fibroblast cultures using TRIzol reagent (Gibco-BRL) according to the manufacturer's directions. Total RNA was resolved on 1.5% agarose-formaldehyde gels (20 μg RNA per lane) and transferred to Hybond-N$^+$ membranes (Amersham). After baking at 80°C for 1 h, each membrane was hybridized with $^{32}$P-dCTP-labelled probes generated using the Oligolabeling Kit (Pharmacia Biotech Inc., Baie d'Urfe, PQ, Canada), and exposed to a Kodak XAR-5 film at –70°C with an intensifying screen. The intensity of the resulting bands (corresponding to *p21*$^{WAF1}$ or *β-actin* transcripts) appearing on the film was quantified by a digitized image analyser (Palcic and Jaggi, 1990).

### RESULTS

Accumulating evidence indicates that many tumour cell lines have bypassed the usual mechanisms by which the cell cycle machinery controls the proliferation of normal cells in response to radiation-activated DNA damage (Nagasawa et al, 1995; Huang et al, 1996; Mirzayans et al, 1997; Olivier et al, 1998). Olivier et al (1998), for example, have reported a relaxed cell cycle arrest in human tumour cell lines harbouring wild-type *p53*. Given that the objective of the experiments described below was to compare the effects of KN62 and wortmannin on cellular radiosensitivity and radiation-activated G1/S and S phase checkpoints, these experiments were performed with diploid normal human fibroblast cultures in order to avoid possible complications associated with the use of tumour cells due to aberrant operation of the cell cycle checkpoint circuitry.

### Radiosensitization of human fibroblasts by wortmannin and KN62

Normal human (GM38) fibroblast cultures were incubated in the presence or absence of a kinase inhibitor for 1 h and exposed to γ radiation. After incubation in the same medium for 10 h, the cells were plated at appropriate densities (100–10 000 cells per 100-mm dish) and allowed to form macroscopic colonies in the absence of the drug. Both kinase inhibitors potentiated radiosensitivity in normal fibroblasts (Figure 1). Following 2 Gy irradiation, for example, clonogenic survival was reduced to 60% in control cultures, 35% in cultures treated with KN62 and 20% in cultures treated with wortmannin.

### Differential effects of wortmannin and KN62 on radiation-activated G1/S checkpoint

To assess the effects of wortmannin and KN62 on G1/S cell cycle arrest, GM38 cultures were incubated with a 10 μM concentration

© 1999 Cancer Research Campaign

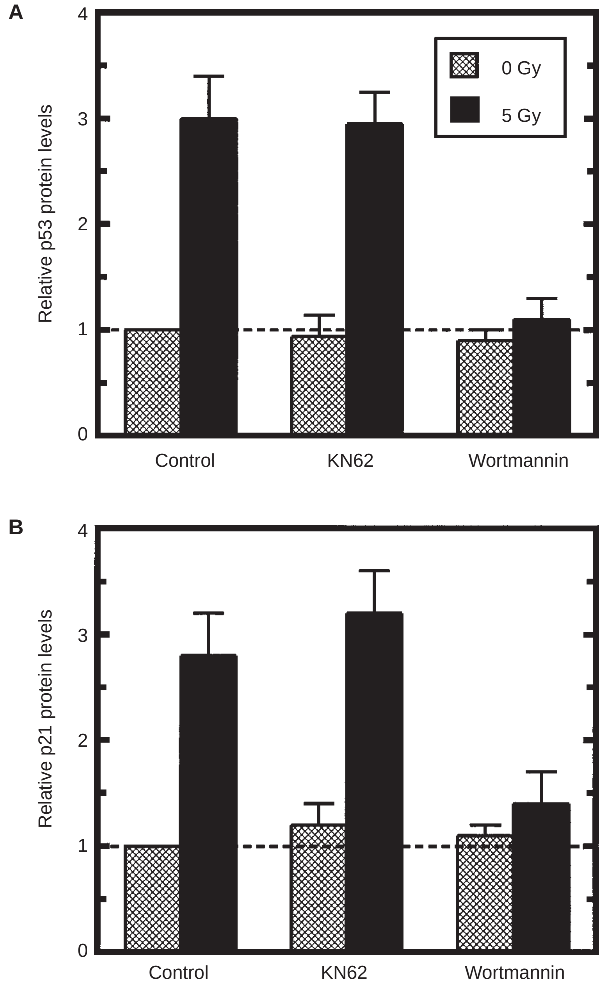

**Figure 3** Effects of wortmannin and KN62 on radiation-induced increases in p53 (**A**) and p21 (**B**) protein levels in GM38 fibroblasts. Cultures were incubated for 1 h with a kinase inhibitor (each at 10 μM) prior to irradiation with 0 or 5 Gy. Following a 3-h incubation in the same medium, the cells were lysed and their p53 and p21 protein contents determined by Western blotting. The data are expressed as normalized p53 (or p21) values for a given treated sample relative to that for the control sample treated with neither radiation nor a kinase inhibitor (set at 1). The means (± s.e.m.) of three determinations are presented

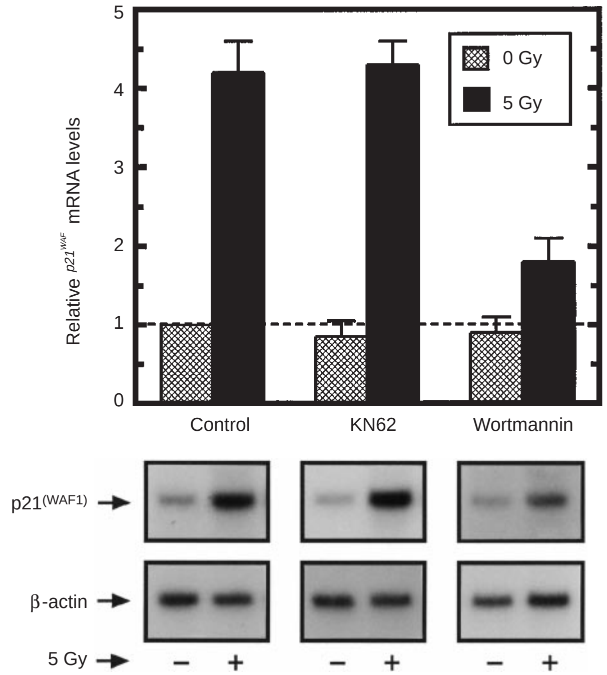

**Figure 4** Effects of wortmannin and KN62 on radiation induction of *p21*$^{WAF1}$ in GM38 fibroblasts. Cultures were treated as described in the legend of Figure 3 and *p21*$^{WAF1}$ mRNA transcripts were quantified by Northern blotting. As a loading control, blots were stripped and reprobed for *β-actin*. Representative blots showing *p21*$^{WAF1}$ and *β-actin* transcripts as well as relative *p21*$^{WAF1}$ mRNA levels determined at 3-h post-irradiation are presented. The densitometric measurements for *p21*$^{WAF1}$ were normalized to those for corresponding *β-actin* mRNA. The data are expressed as relative values for each sample normalized to that of the control sample treated with neither radiation nor a kinase inhibitor (set at 1). The means (± s.e.m.) of three determinations are presented of each drug for 1 h, exposed to 10 Gy of γ radiation (or sham-irradiated) and incubated for various times in the same medium. Tritiated-dThd was added during the last 30 min of the incubation period, after which the cells were processed for autoradiography and the fraction of S phase cells (i.e. those with heavily labelled nuclei) was determined. Under these conditions the ability of human cells to activate the G1/S checkpoint in response to genotoxic stress can be accurately determined (Mirzayans et al, 1995*b*). As expected (Mirzayans et al, 1995*b*), in control (solvent-treated) cultures, the fraction of cells in S phase decreased as a function of time after irradiation and reached ~20% of that in unirradiated cultures by 10 h, indicating the activation of a G1/S checkpoint pathway (Figure 2). Treatment of the fibroblast cultures with wortmannin completely inhibited their ability to activate the G1/S checkpoint (Figure 2), a response similar to that seen in AT cells that were not incubated with a kinase inhibitor (Mirzayans et al, 1995*b*). In contrast to wortmannin, KN62 had no significant effect on the radiation-activated G1/S checkpoint in normal fibroblasts (Figure 2).

### Wortmannin, but not KN62, mitigates the radiation-induced up-regulation of p53 protein and expression of the *p21*$^{WAF1}$ gene

We next determined the effects of wortmannin and KN62 on radiation-induced p53 protein up-regulation and *p21*$^{WAF1}$ expression. To this end, GM38 fibroblasts were incubated in the presence of a kinase inhibitor for 1 h and exposed to 5 Gy (or sham-irradiated). Following incubation in the same medium for 3 h, the cells were lysed and assayed for levels of p53 and p21 proteins as well as *p21*$^{WAF1}$ transcripts. As shown in Figure 3A, radiation exposure resulted in an increase in the amount of p53 protein by ~threefold in control and KN62-treated cultures, but by only 1.2-fold in wortmannin-treated cultures. Radiation exposure also increased the amounts of p21 protein (Figure 3B) and *p21*$^{WAF1}$ transcripts in control GM38 cells (Figure 4); wortmannin inhibited *p21*$^{WAF1}$ mRNA and p21 protein induction by γ rays while KN62 was ineffective (Figures 3B and 4).

### Both wortmannin and KN62 confer radioresistant DNA synthesis on normal human fibroblasts

To compare the effects of KN62 and wortmannin on the radiation-induced S phase checkpoint, GM38 fibroblasts were incubated with an inhibitor for 1 h, exposed to varying doses of γ rays and incubated for 90 min in the same medium; [$^{3}$H]dThd was added to the culture medium during the last 30 min, whereupon the cells

 © 1999 Cancer Research Campaign

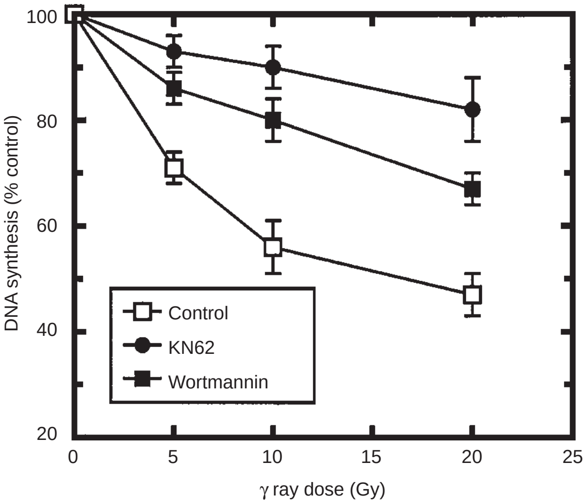

**Figure 5** Effects of wortmannin and KN62 on the extent of inhibition of DNA synthesis induced by γ rays in GM38 fibroblasts. Cultures were incubated in medium containing DMSO (control) or a kinase inhibitor (each at 10 μM) for 1 h prior to irradiation and for 90 min afterwards. Tritiated-dThd was added during the last 30-min of the incubation period and the rate of DNA synthesis was determined as described in Materials and Methods. Bars, s.e.m.

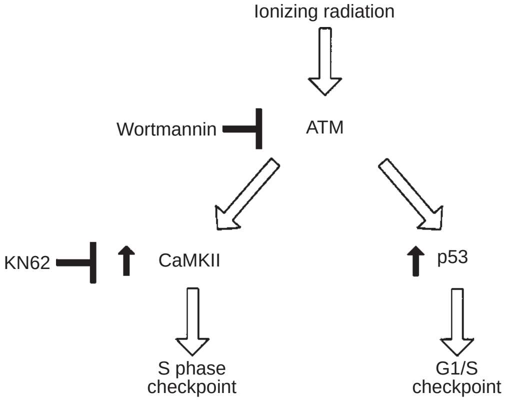

**Figure 7** Proposed mechanisms of the inhibitory effects of wortmannin and KN62 on radiation-induced cell cycle checkpoints. Exposure of normal human fibroblasts to ionizing radiation results in activation of at least two signalling pathways: (i) a p53-dependent pathway which causes a delay in the transition from G1 to S phase (Kastan et al, 1992; Khanna and Lavin, 1993); and (ii) a p53-independent pathway which triggers a transient suppression of replicon initiation and DNA chain elongation in cells in S phase at the time of irradiation (Mirzayans et al, 1995*a*). A KN62-sensitive kinase (i.e. CaMKII; Nakanishi et al, 1992) participates in the S phase checkpoint pathway but not in the p53-dependent pathway, whereas a wortmannin-sensitive kinase (e.g. ATM; Banin et al, 1998) functions upstream in both pathways

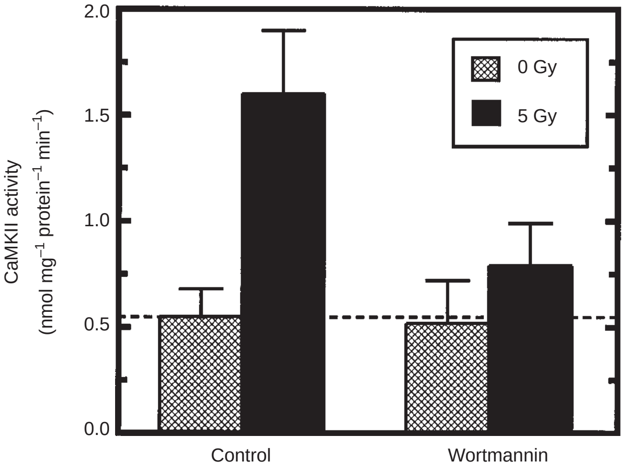

**Figure 6** The influence of wortmannin on the induction of CaMKII activity by γ rays. GM38 fibroblasts were incubated in medium containing either DMSO (control) or 10 μM wortmannin for 1 h. Immediately after γ irradiation (0 or 10 Gy), the cells were lysed and assayed for CaMKII activity as described in Materials and Methods. The means (± s.e.m.) of six determinations from three independent experiments are presented were lysed and the amount of [$^3$H]dThd incorporated into genomic DNA was determined. The results obtained are presented in Figure 5. Irradiation of GM38 fibroblasts, which had been incubated in the absence of a kinase inhibitor, caused a marked reduction in the rate of [$^3$H]dThd incorporation when compared to unirradiated cultures. This reduction in DNA precursor uptake seen at early times post-irradiation reflects a shutdown of the DNA synthesis machinery, with respect to both initiating new replicons and elongating those already in operation (Mirzayans et al, 1995*b*; Painter, 1986). Cultures treated with either KN62 or wortmannin exhibited radioresistant DNA synthesis (Figure 5), a phenotype universally displayed by AT cells (Young and Painter, 1989). Following 10 Gy irradiation, for example, the rate of DNA synthesis decreased by 45% in control (solvent-treated) cultures but only by 20% and 10% in cultures treated with either wortmannin or KN62 respectively (Figure 5).

### Wortmannin mitigates the radiation-induced enhancement of CaMKII activity

We demonstrated recently that exposure of normal human fibroblasts to γ rays resulted in an increase in CaMKII activity, which is required to activate the S phase checkpoint, and further that this response could be mitigated by treatment of cells with KN62 (Famulski et al, submitted). The results in Figure 5 demonstrating radioresistant DNA synthesis in wortmannin-treated normal fibroblasts prompted us to investigate the effect of this drug on radiation induction of CaMKII activity. As shown in Figure 6, radiation exposure resulted in an increase in CaMKII activity by ~threefold in control cultures but only by ~1.5-fold in wortmannin-treated cultures.

### DISCUSSION

In our previous studies, we showed that treatment of normal human fibroblasts with antagonists of calmodulin (i.e. W7, W13) or CaMKII (i.e. KN62) produces an AT-like, radioresistant DNA synthesis phenotype (Mirzayans et al, 1995*a*). Moreover, exposure of normal cells to γ radiation led to a threefold enhancement of CaMKII activity, while AT cells (which exhibit radioresistant DNA synthesis) were defective in this response (Famulski et al, submitted). These results suggest that radiation-induced suppression of DNA synthesis may be mediated through a CaMKII-dependent signalling pathway. In the current study we have shown that, like KN62, wortmannin also confers the radioresistant DNA synthesis phenotype on normal fibroblasts (Figure 5) and mitigates their ability to increase CaMKII activity following radiation exposure (Figure 6). Given that wortmannin is ineffective in

© 1999 Cancer Research Campaign modulating CaMKII activity when added directly to the CaMKII reaction mixture (Nakanishi et al, 1992), we conclude that a wortmannin-sensitive kinase and CaMKII participate in the same signalling pathway controlling suppression of DNA synthesis in irradiated cells, and that the former kinase probably functions upstream of CaMKII in this pathway (also see Figure 7).

We have further demonstrated that wortmannin-treated normal fibroblasts exhibit diminished capacity for both up-regulation of p53 protein (Figure 3A) and expression of the *p21*$^{WAF1}$ gene (Figures 3B and 4) in response to radiation exposure. The treated cultures also fail to activate the G1/S checkpoint post-irradiation (Figure 2), a process known to be controlled by p21 in normal human cells (El-Deiry et al, 1993; Brugarolas et al, 1995). Other groups have found wortmannin to inhibit induction of the DNA-binding activity of wild-type p53 protein in murine cells exposed to ionizing radiation (Price and Youmell, 1996). Taken together, these results suggest that a wortmannin-sensitive kinase functions in the p53 signalling pathway which mediates the G1/S checkpoint in irradiated cells. Contrary to our observation for wortmannin-treated cells, KN62-treated cells responded normally to γ irradiation with respect to up-regulation of p53 protein, expression of the *p21*$^{WAF1}$ gene, and activation of the G1/S checkpoint (Figures 2–4). Therefore, CaMKII, which is required for S phase arrest, does not appear to play a significant role in the p53 signalling pathway that controls the G1 arrest.

In keeping with its ability to inhibit several members of the PI 3-kinase family, wortmannin can potentiate radiosensitivity not only in normal human cells (this study), but also in DNA-PK-deficient cells (Rosenzweig et al, 1997) and in ATM-deficient cells (Rosenzweig et al, 1997). In addition, wortmannin has been reported to enhance radiosensitivity in human tumour cell lines harbouring wild-type or mutant *p53* gene (Price and Youmell, 1996). Thus, although PI 3-kinase and related proteins represent excellent targets for the radiosensitization of malignant cells, the clinical utility of wortmannin may be limited owing to its ability to inhibit multiple radiation-responsive pathways. Our current findings, however, demonstrating potentiation of radiosensitivity in human cells by KN62 and wortmannin (Figure 1), warrant further studies to explore the clinical ramifications of pharmacological inhibitors of CaMKII (e.g. KN62) and specific PI 3-kinase-related proteins as antineoplastic drugs.

Several reports have demonstrated an inverse correlation between the susceptibility of a variety of mammalian cell cultures to killing by genotoxic agents and their ability to activate the S phase checkpoint (Wang and Iliakis, 1992; Jung et al, 1995; Mirzayans et al, 1995*b*; Morgan et al, 1997; Shao et al, 1997). Accordingly, pharmacological abrogation of DNA damage-induced cell cycle checkpoints (e.g. S phase arrest) by antagonists of protein kinases has been proposed as an effective strategy for selectively enhancing the cytotoxicity of therapeutic agents (Shao et al, 1997). In light of this, we speculate that potentiation of radiosensitivity by wortmannin and KN62 (Figure 1) may in part be associated with abrogation of the radiation-induced S phase arrest (Figure 5). Wortmannin is shown here to produce a greater impact on radiation cytotoxicity than KN62 (Figure 1). This latter observation is not surprising because KN62 specifically inactivates CaMKII (Tokumitsu et al, 1990), while wortmannin not only influences the radiation induction of CaMKII activity (Figure 6), but also inhibits ATM, ATR and DNA-PK, all of which play vital roles in the cellular responses to genotoxic stresses (Powis et al, 1994; Hartley et al, 1995; Banin et al, 1998; Cliby et al, 1998).

In summary, we have demonstrated that CaMKII, which is known to be involved in the signalling pathway that mediates inhibition of DNA synthesis in normal human fibroblasts exposed to γ radiation (Mirzayans et al, 1995*a*), does not play a significant role in the p53-mediated pathway controlling the G1/S transition. These results give credence to our previous findings which implicated distinct mechanisms in the radiation-activated S phase and G1/S checkpoints (also see Figure 7). A wortmannin-sensitive kinase, on the other hand, appears to function upstream in both the S phase and G1/S checkpoints (Figure 7). The wortmannin-sensitive kinase proposed to function in these two pathways is most likely ATM, because radiation responsive cell cycle checkpoints are impaired in cells from AT patients (Beamish and Lavin, 1994), and because among all wortmannin-sensitive kinases examined to date only ATM participates in the radiation-induced S phase arrest (Komatsu et al, 1993; Meyn, 1995; Cliby et al, 1998). Our results also suggest that mitigation of the S phase checkpoint may underlie, at least in part, the potentiation of radiosensitivity evoked by both wortmannin and KN62 in normal human cells.

### ACKNOWLEDGEMENTS

The authors wish to thank Drs Malcolm C Paterson and Konrad Famulski for helpful comments. This work was supported by the National Cancer Institute of Canada with funds from the Canadian Cancer Society and the Terry Fox Foundation.

### REFERENCES

Banin S, Moyal L, Shieh SY, Taya Y, Anderson CW, Cheesa L, Smorodinsky NI, Prives C, Reiss Y, Shiloh Y and Ziv Y (1998) Enhanced phosphorylation of p53 by ATM in response to DNA damage. *Science* **281**: 1674–1677

Beamish H and Lavin MF (1994) Radiosensitivity in ataxia-telangiectasia: anomalies in radiation-induced cell cycle delay. *Int J Radiat Biol* **65**: 175–184

Brugarolas J, Chandrasekaran C, Gordon JI, Beach D, Jacks T and Hannon GJ (1995) Radiation-induced cell cycle arrest compromised by p21 deficiency. *Nature* **377**: 552–557

Cliby WA, Roberts CJ, Cimprich KA, Stringer CM, Lamb JR, Schreiber SL and Friend SH (1998) Overexpression of a kinase-inactive ATR protein causes sensitivity to DNA-damaging agents and defects in cell cycle checkpoints. *EMBO J* **17**: 159–169

El-Deiry WS, Tokino T, Velculescu VE, Levy DB, Parsons R, Trent JM, Lin D, Mercer WE, Kinzler KW and Vogelstein B (1993) *WAF1*, a potent mediator of p53 tumor suppressor. *Cell* **75**: 817–825

Hartley KO, Gell D, Smith GCM, Zhang H, Divecha N, Connelly MA, Admon A, Lees-Miller SP, Anderson CW and Jackson SP (1995) DNA-dependent protein kinase catalytic subunit: a relative of phosphatidylinositol 3-kinase and the ataxia telangiectasia gene product. *Cell* **82**: 849–856

Huang H, Li C-Y and Little JB (1996) Abrogation of p53 function by transfection of HPV16 E6 gene does not enhance resistance of human tumour cells to ionizing radiation. *Int J Radiat Biol* **70**: 151–160

Jung M, Zhang Y, Lee S and Dritschilo A (1995) Correction of radiation sensitivity in ataxia telangiectasia cells by truncated IκB-α. *Science* **268**: 1619–1621

Kastan MB, Zhan Q, El-Deiry WS, Carrier F, Jacks T, Walsh WV, Plunkett BS, Vogelstein B and Fornace Jr AJ (1992) A mammalian cell cycle checkpoint pathway utilizing p53 and *GADD45* is defective in ataxia-telangiectasia. *Cell* **71**: 587–597

Khanna KK and Lavin MF (1993) Ionizing radiation and UV induction of p53 protein by different pathways in ataxia-telangiectasia cells. *Oncogene* **8**: 3307–3312

Komatsu K, Yoshida M and Okumura Y (1993) Murine SCID cells complement ataxia-telangiectasia cells and show a normal post-irradiation response to DNA synthesis. *Int J Radiat Biol* **63**: 725–730

Lavin MF (1993) Biochemical defects in ataxia-telangiectasia. In: *Ataxia-telangiectasia*. RA Gatti and RB Painter (eds), NATO ASI series H, Vol 77, pp. 234–255. Springer-Verlag: Berlin.

 © 1999 Cancer Research Campaign

Mayford M, Wang J, Kandel ER and O'Dell TJ (1995) CaMKII regulates the frequency-response function of hippocampal synapses for the production of both LTD and LTP. *Cell* **81**: 891–904

Meyn MS (1995) Ataxia-telangiectasia and cellular responses to DNA damage. *Cancer Res* **55**: 5991–6001

Mirzayans R, Smith BP and Paterson MC (1989) Hypersensitivity to cell killing and faulty repair of 1-β-D-arabinofuranosylcytosine-detectable sites in human (ataxia-telangiectasia) fibroblasts treated with 4-nitroquinoline 1-oxide. *Cancer Res* **49**: 5523–5529

Mirzayans R, Famulski KS, Enns L, Fraser M and Paterson MC (1995*a*) Characterization of the signal transduction pathway mediating γ ray-induced inhibition of DNA synthesis in human cells: indirect evidence for involvement of calmodulin but not protein kinase C or p53. *Oncogene* **11**: 1597–1605

Mirzayans R, Aubin RA, Bosnich W, Blattner WA and Paterson MC (1995*b*) Abnormal pattern of post-γ-ray DNA replication in radioresistant fibroblast strains from affected members of a cancer-prone family with Li–Fraumeni syndrome. *Br J Cancer* **71**: 1221–1230

Mirzayans R, Enns L and Paterson MC (1997) Inhibition of DNA synthesis and G1/S-phase transition in normal human fibroblasts elicited by a heat-labile *trans*-acting factor in gamma-irradiated HeLa cell extracts. *Radiat Res* **147**: 13–21

Morgan SE, Lovly C, Pandita TK, Shiloh Y and Kastan MB (1997) Fragments of ATM which have dominant-negative or complementary activity. *Mol Cell Biol* **17**: 2020–2029

Nagasawa H, Li Y, Maki CG, Imrich AC and Little JB (1995) Relationship between radiation-induced G1 phase arrest and p53 function in human tumor cells. *Cancer Res* **55**: 1842–1846

Nakanishi S, Kakita S, Takahashi I, Kawahara K, Tsukuda E, Sano T, Yamada K, Yoshida M, Kase H, Matsuda Y, Hashimoto Y and Nonomura Y (1992) Wortmannin, a microbial product inhibitor of myosin light chain kinase. *J Biol Chem* **267**: 2157–2163

Olivier M, Bautista S, Valles H and Theillet C (1998) Relaxed cell-cycle arrest and propagation of unrepaired chromosomal damage in cancer cell lines with wild-type *p53*. *Mol Carcinogenesis* **23**: 1–12

Painter RB (1986) Inhibition of mammalian cell DNA synthesis by ionizing radiation. *Int J Radiat Biol* **49**: 771–781

Palcic B and Jaggi B (1990) Image cytometry system for morphometric measurements of live cells. In: *Bioinstrumentation: Research, Developments and Applications*, DL Wise (ed), pp. 923–991. Butterworth: Stoneham, MA.

Powis G, Bonjouklian R, Berggren MM, Gallegos A, Abraham R, Ashendel C, Zalkow L, Matter WF, Dodge J, Grindey G and Vlahos CJ (1994) Wortmannin, a potent and selective inhibitor of phosphatidylinositol-3-kinase. *Cancer Res* **54**: 2419–2423

Price BD and Youmell MB (1996) The phosphatidylinositol 3-kinase inhibitor wortmannin sensitizes murine fibroblasts and human tumor cells to radiation and blocks induction of *p53* following DNA damage. *Cancer Res* **56**: 246–250

Rosenzweig KE, Youmell MB, Palayoor ST and Price BD (1997) Radiosensitization of human tumor cells by the phosphatidylinositol 3-kinase inhibitors wortmannin and LY294002 correlates with inhibition of DNA-dependent protein kinase and prolonged $G_2$-M delay. *Clin Cancer Res* **3**: 1149–1156

Savitsky K, Bar-Shira A, Gilad S, Rotman G, Ziv Y, Vanagaite L, Tagle DA, Smith S, Uziel T, Sfez S, Ashkenazi M, Pecker I, Frydman M, Harnik R, Patanjali SR, Simmons A, Clines GA, Sartiel A, Gatti RA, Chessa L, Sanal O, Lavin MF, Jaspers NGJ, Taylor AMR, Arlett CF, Miki T, Weissman SM, Lovett M, Collins FS and Shiloh Y (1995) A single ataxia telangiectasia gene with a product similar to Pl-3 kinase. *Science* **268**: 1749–1753

Sedgwick RP and Boder E (1991) Ataxia-telangiectasia. *Handb Clin Neurol* **16**: 347–423

Shao RG, Cao CX, Shimizu J, O'Conner PM, Kohn KW and Pommier Y (1997) Abrogation of an S-phase checkpoint and potentiation of camptothecin cytotoxicity by 7-hydroxystaurosporine (UCN-01) in human cancer cell lines, possibly influenced by p53 function. *Cancer Res* **57**: 4029–4035

Taylor AMR (1998) What has the cloning of the *ATM* gene told us about ataxia telangiectasia? *Int J Radiat Biol* **73**: 365–371

Tokumitsu H, Chijiwa T, Hagiwara M, Mizutani A, Terasawa M and Hidika H (1990) KN-62, 1-[*N*,*O*-Bis(5-isoquinolinesulfonyl)-*N*-methyl-L-tyrosyl]-4-phenylpiperazine, a specific inhibitor of $Ca^{2+}$/calmodulin-dependent protein kinase II. *J Biol Chem* **265**: 4315–4320

Wang Y and Iliakis G (1992) Prolonged inhibition by X-rays of DNA synthesis in cells obtained by transfection of primary rat embryo fibroblasts with oncogenes H-*ras* and v-*myc*. *Cancer Res* **52**: 508–514

Young BR and Painter RB (1989) Radioresistant DNA synthesis and human genetic diseases. *Hum Genet* **82**: 113–117

© 1999 Cancer Research Campaign 
